# Association of body surface scanner-based abdominal volume with parameters of the Metabolic Syndrome and comparison with manually measured waist circumference

**DOI:** 10.1038/s41598-020-66095-6

**Published:** 2020-06-09

**Authors:** Lina Jaeschke, Astrid Steinbrecher, Guido Hansen, Stefan Sommer, Carolin Adler, Jürgen Janke, Tobias Pischon

**Affiliations:** 1 Molecular Epidemiology Research Group, Max Delbrück Center for Molecular Medicine in the Helmholtz Association (MDC), Berlin, Germany; 2 Avalution GmbH, Kaiserslautern, Germany; 3 Charité – Universitätsmedizin Berlin, Berlin, Germany; 4 German Center for Cardiovascular Research (DZHK), partner site Berlin, Berlin, Germany; 5 MDC/BIH Biobank, Max Delbrück Center for Molecular Medicine (MDC) and Berlin Institute of Health (BIH), Berlin, Germany

**Keywords:** Metabolic disorders, Physical examination, Disease prevention, Epidemiology, Risk factors

## Abstract

To investigate abdominal volume determined by a new body scanner algorithm as anthropometric marker for Metabolic Syndrome (MetS) and its parameters compared to manually measured waist circumference (WC), we performed body scans in 411 participants (38% men, 20-81 years). WC and triglyceride, HDL-cholesterol, and fasting glucose concentrations, and blood pressure were assessed as MetS parameters. We used Spearman correlations and linear regression to investigate associations and goodness-of-fit (R², BIC) of abdominal volume and WC with MetS parameters, and logistic regression to analyse the discriminative power of WC and abdominal volume to assess likelihoods of MetS components and MetS. Correlations with triglyceride, HDL-cholesterol, and glucose concentration were slightly stronger for abdominal volume (r; 0.32, −0.32, and 0.34, respectively) than for WC (0.28, −0.28, and 0.29, respectively). Explained variances in MetS parameters were slightly higher and goodness-of-fit slightly better for abdominal volume than for WC, but differences were small. Exemplarily, glucose levels were 0.28 mmol/L higher (R² = 0.25; BIC = 945.5) per 1-SD higher  WC, and 0.35 mmol/L higher (R² = 0.28; BIC = 929.1) per 1-SD higher abdominal volume. The discriminative power to estimate MetS components was similar for WC and abdominal volume. Our data show that abdominal volume allows metabolic characterization comparable to established WC.

## Introduction

Abdominal obesity, characterized by increased visceral fat in the abdomen, is an important risk factor in the aetiology of chronic diseases^1–4^. It is the main component of the Metabolic Syndrome (MetS), a cluster of metabolic disorders including an increased waist circumference (WC), elevated triglyceride (TG) and fasting glucose concentrations, reduced high-density lipoprotein cholesterol (HDL-C) concentrations, and high blood pressure^5^. In 2014, the prevalence of MetS was estimated to be around 25% worldwide, with a steady increase over the past decades^1,6–8^. So far, assessing abdominal obesity primarily relies on WC measurements by tape^9^, but this only allows an assessment in two spatial horizontal dimensions. Contrary, measurement of abdominal volume would be expected to give a more accurate estimation of visceral fat.

Three-dimensional (3D) body surface scanners (BS) objectively assess anthropometry on a large scale in short time. Being originally developed for the clothing industry, these devices automatically capture a 3D picture of the human body and allow determining more than 150 anthropometric measures. We have recently shown that BS may also be used to measure WC and overall body volume with good validity and reliability^10,11^. However, it is unclear to what extent BS may also be used to measure abdominal volume and to characterize metabolic alterations.

The aim of the present study was, therefore, to investigate, how good abdominal volume, automatically determined using a newly derived BS algorithm, allows metabolic characterization in an epidemiological setting compared to the established manually measured WC. For this purpose, we examined correlations as well as strengths of associations and goodness-of-fit of WC and abdominal volume with MetS parameters. We further investigated the discriminative power of WC and abdominal volume to estimate the likelihood of presence of MetS components (i.e., MetS parameters outside the reference range) and MetS itself.

## Materials and methods

### Study population

Data for the present cross-sectional MetScan study were collected between February 2016 and June 2017 at the Max Delbrück Center for Molecular Medicine in the Helmholtz Association, Berlin, Germany, Molecular Epidemiology Research Group. In total, 516 men and women were recruited as a convenience sample using institutional and university mailing lists, and newspaper and public postings following a standardized recruitment protocol. Inclusion criteria were age 18–79 years, German language skills, and ability to give informed consent. Exclusion criteria were inability to perform BS measurement (e.g., inability to stand free) and any condition affecting body shape (i.e., casts, amputations, pregnancy).

The study protocol was approved by the ethics committee of the Charité – Universitätsmedizin Berlin and the local data protection officer. All investigations were carried out in accordance with the relevant guidelines and regulations, and written informed consent was obtained from all participants before inclusion.

### Data collection

On site, all participants completed a personal computer-assisted interview on sex, age, and history of diabetes mellitus and dyslipidaemia ever diagnosed by a physician.

Following a five minutes rest, three sitting blood pressure measurements with 2-minute intervals were performed using the gauge HEM 705IT (OMRON, Mannheim, Germany) and a cuff suitable for the upper arm circumference. We excluded the first measurement and calculated the mean of the second and third systolic and diastolic blood pressure measurement^12^.

Participants provided fasting blood samples (fasting state >8 hours), and we determined TG, HDL-C, glucose, and HbA1c concentrations. All laboratory analyses were performed by the hospital Laborverbund Brandenburg-Berlin GmbH (Berlin, Germany).

Anthropometry was taken manually by trained personnel according to guidelines of the World Health Organization^9^. With an accuracy of one decimal place, body height (cm, stadiometer SECA 285, Hamburg, Germany), body weight (kg, bioelectrical impedance analysis device SECA mBCA 515, Hamburg, Germany), and WC (cm, measuring tape SECA 201, Hamburg, Germany) were measured. We calculated body mass index (BMI) as body weight divided by the square of body height (kg/m²) and waist-to-height ratio (WHtR).

For 3D measurement, the BS Vitus Smart XXL and the AnthroScan Professional software were used (both Avalution GmbH, Kaiserslautern, Germany). Based on four eye-safe lasers, eight cameras, and optical triangulation, the BS generates a 3D point cloud depicting the body surface within 12 seconds. Following international norms^13^, ≥150 anthropometric measures are determined from the 3D picture (accuracy, ±1 mm; density, 27 points/cm²; around 500,000 points/scan). The BS was calibrated daily using a cylinder tube of defined height and circumference according to the manufacturer’s instructions. Participants were asked to undress up to the underwear (tight-fitting, unpadded), to remove jewellery or eyeglasses, and to put on bathing caps covering hair. They were instructed to stand upright in the BS with head positioned according to the Frankfort Horizontal plane^9^, legs hip-wide apart, arms relaxed without body contact, if possible, and hands making a fist with thumbs outside showing forward. Breathing should be normal. After each scan, 3D pictures were immediately visually quality checked by study personnel, i.e., for image errors or artefacts, or deviations from the standard posture. If quality was insufficient, scanning was repeated with a maximum of three attempts; otherwise, scanning was skipped.

**Figure 1:**
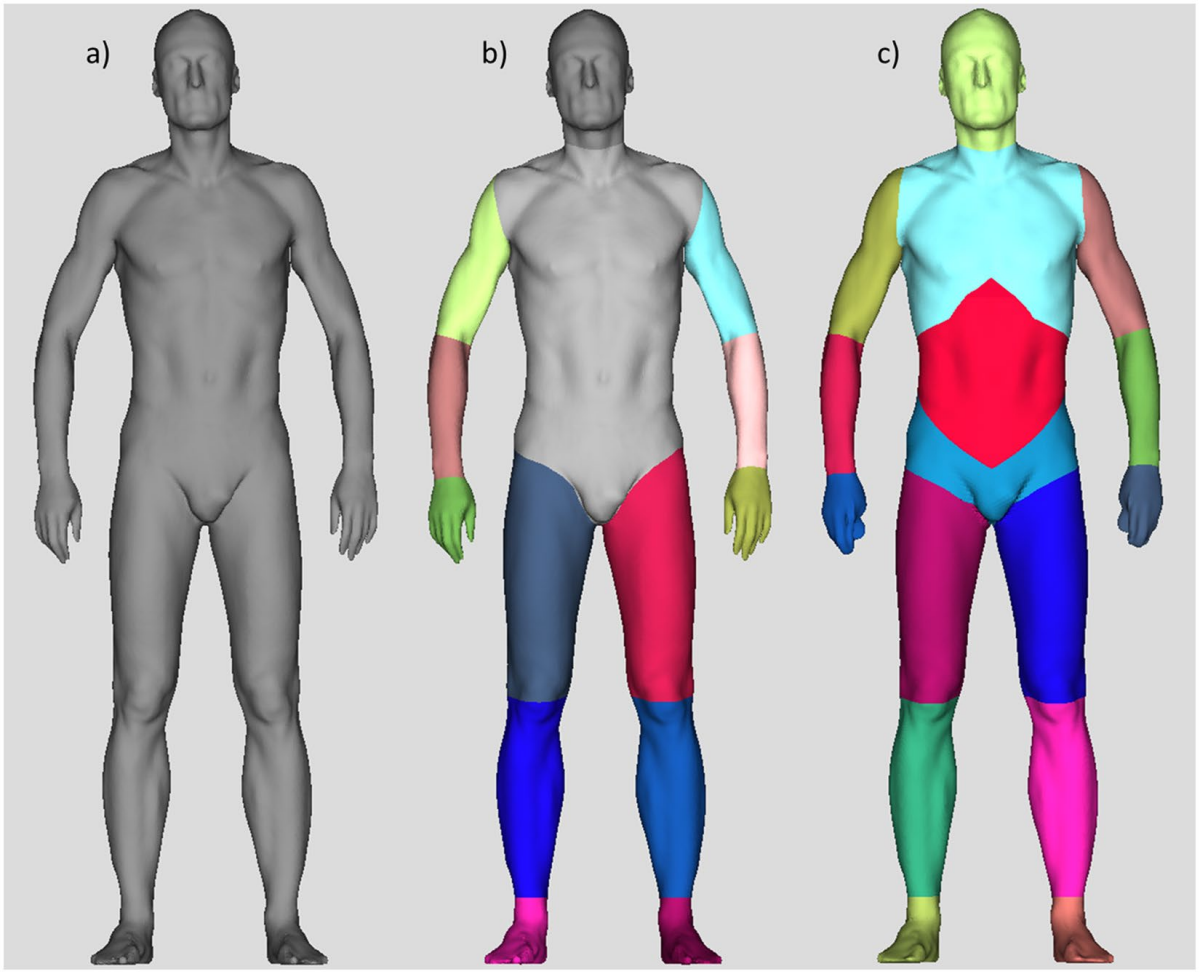
Automated determination of different body volumes based on 3D body scans. The 3D body scanner allows determination of the overall body volume (panel a) and of different body volume parts (panel b), including the torso volume (light grey, panel b). Based on scans with hemispherical markers attached to the participants’ skin to palpable anatomical limit points of the abdominal volume, a new algorithm was derived, now enabling automated determination of abdominal volume (red, panel c).

Besides numerous anthropometric measures, the BS enables volumetric determination of overall volume and volume of head, hands, lower/upper arms and legs, feet, and torso (Fig. 1, panel a,b). So far, a specific determination of abdominal volume has not been possible. In the course of a cooperation project, we therefore developed an algorithm to determine abdominal volume as a potential alternative anthropometric risk measure (Fig. 1, red, panel c).

**Figure 2:**
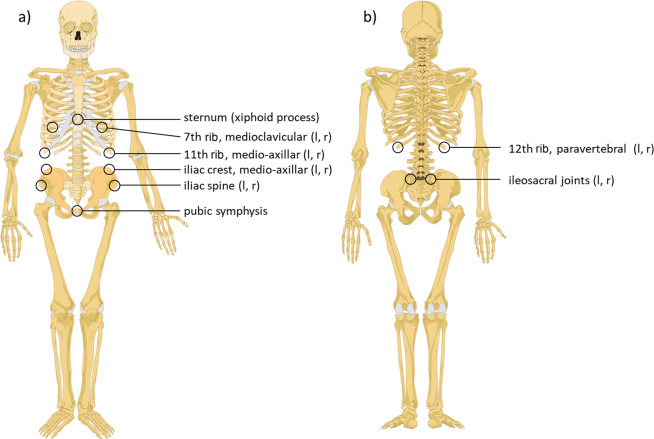
Positions of hemispherical markers attached to the skin of participants bounding the abdominal volume for the development of the 3D body scan algorithm for automated determination of the abdominal volume. Fourteen hemispherical markers were attached to the front (panel a) and rear (panel b) part of the participants’ skin to represent internal anatomical structures bounding the abdominal volume. Based on scan pictures taken with these markers accordingly attached to the skin, the 3D body scan algorithm for automated determination of abdominal volume was developed. Figure modified based on graphs obtained from Wikimedia commons, front: https://commons.wikimedia.org/wiki/File:Human_skeleton_front_en.svg#/media/File:Human_skeleton_front_-_no_labels.svg; rear: https://commons.wikimedia.org/wiki/File:Human_skeleton_back_en.svg#/media/File:Human_skeleton_back_en.svg. l, left; r, right.

For this purpose, prior to each measurement, small hemispherical markers (‘landmarks’) were attached to the participants’ skin reflecting palpable anatomical limit points of the abdominal volume, including seven landmarks cranially according to the boundary of the diaphragm and seven landmarks caudally according to the boundary of the pelvis (Fig. 2). Landmark placement was done only for development of the later fully automatic system. For the development of the abdominal volume algorithm, a subsample of about 100 marked scans was transferred to our cooperation partner Avalution GmbH (Kaiserslautern, Germany). During the learning phase, the algorithm took the raw point cloud of the scans and converted it into a homologous surface representation. Having this together with the attached landmark positions for each scan, the mean homologous coordinate for each landmark was learned. The landmarks were taken to extract boundary lines to isolate the sub segment of the abdomen in a geometrical representation (surface model). During the operation phase, the same scan conversion takes place and since landmark positions now are known, the individual abdominal volume can be computed fully automatically without any additional treatment (Fig. 1, red, panel c). The newly developed algorithm was implemented in the BS software and abdominal volume (L) was determined for all participants.

We calculated the abdominal-to-overall-volume ratio (AOR) and the abdominal-volume-to-height ratio (AHtR, L/cm). Height measure was obtained from manual measurement^10^.

### Classification of metabolic parameters to assess MetS components

Metabolic components were classified according to the Harmonized model as follows^5^: elevated TG concentration (≥1.7 mmol/L); decreased HDL-C concentration (men, <1.0 mmol/L; women, <1.3 mmol/L); elevated blood pressure (mean systolic blood pressure ≥130 mmHg or mean diastolic blood pressure ≥85 mmHg or reported diagnosis of hypertension); elevated fasting glucose concentration (≥5.6 mmol/L or, if glucose was missing, HbA1c concentration ≥38.8 mmol/mol, or reported diagnosis of diabetes). MetS was defined as having three or more of the following five criteria: elevated WC (men, ≥94 cm; women, ≥80 cm), elevated TG, elevated fasting glucose, and decreased HDL-C concentration, or elevated blood pressure^5^.

### Statistical analyses

We excluded participants not being in fasting state (n = 35) or without any information from laboratory analysis due to unsuccessful or insufficient blood draw (n = 10). We also excluded participants who did not take part in BS measurement due to technical issues or inability of participants to perform the procedure (n = 13). Further, participants were excluded if the quality of the scan picture taken was not satisfactory regarding abdominal volume (n = 56). Some participants met multiple exclusion criteria. Thus, finally, 411 participants (157 men, 254 women) were included in the present analyses.

For some included participants, however, single MetS parameters were not determined (missing number: systolic and diastolic blood pressure, HDL-C and TG concentration: 1; HbA1c concentration: 3; fasting glucose concentration: 12). Missing values were imputed using the sex-specific median of the respective variable for the correlation and linear regression analyses.

For some participants, fasting glucose, HbA1c, or both concentrations were missing. Thus, if glucose concentration was missing, but the information on HbA1c concentration was available, we used the latter to classify participants’ glucose status^5^. If both fasting glucose and HbA1c concentration were missing, we imputed the missing glucose value as described and classified participants accordingly^5^.

Descriptive data, manual anthropometry, blood pressure, and frequency of MetS components are given as absolute and relative figures or median and interquartile range (IQR). Laboratory and BS parameters are given as geometric mean (GM) and 95% confidence interval (CI).

First, we investigated the association of abdominal volume compared to WC with continuous MetS parameters, i.e., TG, HDL-C, and glucose concentration, and systolic blood pressure (SBP), using Spearman partial correlations adjusted for sex, age, and body height. Similarly, we also investigated the association of BMI and WHtR as well as AOR and AHtR with the continuous MetS parameters.

We performed linear regression analyses with robust variance estimation^14^ and logistic regression analyses to investigate the strength of the association of abdominal volume compared to WC with continuous MetS parameters and presence or absence of MetS components, respectively. Concentrations of TG, HDL-C, and fasting glucose, as well as SBP and presence or absence of MetS components, respectively, were included as single outcomes, and WC or abdominal volume as independent variable, with adjustment for sex, age, and body height. In both linear and logistic regression analyses, we also investigated BMI and WHtR as well as AOR and AHtR as independent variables. We determined ß-coefficients with corresponding p-values and odds ratios (OR) with corresponding 95% CI, respectively, as measures of the strength of the association, R², reflecting the proportion of explained compared to the total variance, and the Bayes Information Criterion (BIC) as goodness-of-fit criterion for not nested models^15^. To assess the discriminative power of abdominal volume compared to WC to estimate presence or absence of MetS components, we further calculated c-statistics as goodness-of-fit criteria in the logistic models. c-statistics are equivalent to the area under the receiver operating characteristic (ROC) curve, representing an overall measure of classification accuracy, i.e., the probability to distinguish between cases and non-cases^16^. Lower values of BIC and higher values of c-statistics indicate better model fit^16^. All association measures referred to a 1-SD change in the respective anthropometric parameter. In further linear analyses, we conducted the same respective analyses with both WC and abdominal volume included as independent variables in joint models. To investigate how good abdominal volume enables detection of MetS itself, we additionally ran a logistic regression with MetS as outcome and abdominal volume as independent variable, while adjusting for sex, age, and body height, referring to a 1-liter change in abdominal volume.

Finally, we performed ROC curves stratified by sex, with MetS components and MetS as outcome and abdominal volume as independent variable. We derived areas under the curve as well as the respective model’s intercept and regression coefficient for abdominal volume to calculate optimal cutoffs for abdominal volume to assess single MetS components and MetS with highest differentiating ability, i.e., high sensitivity and specificity, as evaluated by the Youden Index^17–19^: sensitivity + specificity-1. Median sex-stratified cutoffs and frequency of participants having an abdominal volume above this abdominal volume limit were calculated.

We tested for sex differences by including interaction terms of sex with WC and abdominal volume, respectively.

### Additional analyses

In additional analyses, we excluded participants with single missing MetS parameters instead of imputation, and repeated all analyses (participants included, N = 397). We also performed all analyses for WC divided by the square of body height (WHt²R, 1/cm) and abdominal volume divided by the square of body height (AHt²R, L/cm²)^20,21^. In addition, we tested for differences between included and excluded participants using unpaired t-tests or Mann-Whitney U tests (for continuous variables) and Chi-Square tests (for discrete variables).

P-values presented are two-tailed, with p < 0.05 considered statistically significant. Analyses were performed using SAS® Enterprise Guide® (version 4.3; SAS Institute Inc., Cary, NC).

## Results

Men and women had a median age of 56.0 years (IQR, 41.6 to 68.7) and 52.6 years (35.1 to 63.9), respectively (Table 1). Manually measured WC was 95.5 cm (88.0 to 104.2) in men and 83.5 cm (74.3 to 93.3) in women, and BS-based abdominal volume was 13.6 L (13.1 to 14.2) and 9.3 L (9.0 to 9.6), respectively. MetS was present in 44.6% of men and 28.0% of women. There were no differences in characteristics, when comparing included and excluded participants (data not shown), with exception of the frequency of elevated glucose levels that was higher in included than excluded participants (58.2% versus 45.5%).

**Table 1:** Basic characteristics of participants of MetScan, 2016-2017, total (N = 411).

	men (n = 157)		women (n = 254)	
	median	IQR	median	IQR
age, years	56.0	(41.6, 68.7)	52.6	(35.1, 63.9)
height, cm	178.9	(174.1, 183.5)	166.1	(161.1, 170.0)
WC, cm	95.5	(88.0, 104.2)	83.5	(74.3, 93.3)
BMI, kg/m²	25.9	(24.0, 28.5)	24.7	(21.8, 28.3)
WHtR	0.53	(0.49, 0.59)	0.50	(0.44, 0.57)
SBP, mmHg	129.5	(119.5, 139.0)	117.0	(108.0, 127.5)
DBP, mmHg	79.0	(74.0, 84.5)	73.5	(67.5, 80.0)
	**GM**	**95% CI**	**GM**	**95% CI**
TG, mmol/l	1.22	(1.14, 1.31)	1.07	(1.01, 1.13)
HDL-C, mg/dl	1.36	(1.32, 1.40)	1.64	(1.60, 1.68)
glucose, mmol/l	5.97	(5.85, 6.09)	5.59	(5.51, 5.68)
overall volume, L	85.1	(82.8, 87.4)	71.1	(69.6, 72.6)
abdominal volume, L	13.6	(13.1, 14.2)	9.3	(9.0, 9.6)
AOR	0.160	(0.157, 0.163)	0.131	(0.129, 0.133)
AHtR, L/cm	0.076	(0.073, 0.079)	0.056	(0.054, 0.058)
	**%**		**%**	
diabetes mellitus^a^	7.6		5.1	
dyslipidaemia^a^	28.0		32.3	
MetS	44.6		28.0	
elevated WC^b^	54.1		57.1	
elevated TG^c^	23.6		13.0	
reduced HDL-C^d^	4.5		7.9	
elevated blood pressure^e^	58.6		39.4	
elevated glucose^f^	75.8		47.2	

AHtR, abdominal-volume-to-height ratio; AOR, abdominal-to-overall-volume ratio; BMI, body mass index; DBP, diastolic blood pressure; GM, geometric mean; HDL-C, high-density lipoprotein cholesterol; IQR, interquartile range; MetS, Metabolic Syndrome; SBP, systolic blood pressure; TG, triglycerides; WC, waist circumference; WHtR, waist-to-height ratio; 95% CI, 95% confidence interval.

Information on age and history of diabetes and dyslipidaemia was derived from self-reports during a personal interview; manual anthropometric and 3D body scan measures, blood pressure as well as fasting blood samples were taken by trained personnel

^a^self-reported physician-diagnosed disease

^b^men, ≥94 cm; women, ≥80 cm^5^

^c^ ≥ 1.7 mmol/L^5^

^d^men, <1.0 mmol/L; women, <1.3 mmol/L^5^

^e^systolic blood pressure ≥130 mmHg or mean diastolic blood pressure ≥85 mmHg (mean of the last two out of three sitting blood pressure measurements) or reported diagnosis of hypertension^5^

^f^ ≥ 5.6 mmol/L (or, if information on glucose is missing, HbA1c ≥ 38.8 mmol/mol) or reported medical history of diabetes^5^.

### Correlation analyses

Table 2 summarizes the correlation of WC and abdominal volume with TG, HDL-C, and fasting glucose concentration, and SBP. All correlations were highly significant (p < 0.0001). Overall, correlations with MetS parameters tended to be slightly stronger for abdominal volume than for WC, except for SBP, although the differences in the correlation coefficients of WC versus abdominal volume were small.

**Table 2:** Spearman partial correlation of manually measured waist circumference and body scanner-based abdominal volume with parameters of the Metabolic Syndrome^1^, total (N = 411).

anthropometric measure	TG, mmol/L		HDL-C, mmol/L		SBP, mmHg		glucose, mmol/L	
	r	p	r	p	r	p	r	p
**manual measure**								
WC, cm	0.28	<0.0001	−0.28	<0.0001	0.25	<0.0001	0.29	<0.0001
**body scan measure**								
abdominal volume, L	0.32	<0.0001	−0.32	<0.0001	0.24	<0.0001	0.34	<0.0001

HDL-C, high-density lipoprotein cholesterol; SBP, systolic blood pressure; TG, triglycerides; WC, waist circumference

^1^Model adjusted for adjusted for age, sex, and body height (manual measurement).

### Linear regression analyses

The multivariable association of WC and abdominal volume with MetS parameters is shown in Table 3. Generally, the model fit of the association with MetS parameters was slightly better for abdominal volume than for WC, i.e., standardized β-coefficients and R² were slightly higher and BIC was slightly lower, except for SBP. Nevertheless, differences between WC and abdominal volume were small. For example, a 1-SD difference in WC was associated with a 0.28 mmol/L difference in fasting glucose concentration (p < 0.0001), with 25% of the total variance in glucose concentration explained by WC (R² = 0.25); BIC was 969.6. In contrast, a 1-SD difference in abdominal volume was related to a 0.35 mmol/L difference in glucose concentration (p < 0.0001; R² = 0.28; BIC = 953.2). All associations were highly significant (all p-values <0.0001; except for SBP and abdominal volume, p = 0.0002). There were no significant sex differences in these associations (data not shown).

**Table 3:** Association of manually measured waist circumference and body scanner-based abdominal volume with parameters of the Metabolic Syndrome, total (N = 411)^1^.

anthropometric measure	TG, mmol/L				HDL-C, mmol/L				SBP, mmHg				glucose, mmol/L			
	β	p	R²	BIC	β	p	R²	BIC	β	p	R²	BIC	β	p	R²	BIC
**manual measure**																
WC, per SD	0.19	<0.0001	0.11	759.8	−0.10	<0.0001	0.26	157.2	3.84	<0.0001	0.31	3310.6	0.28	<0.0001	0.25	969.6
**body scan measure**																
abdominal volume, per SD	0.23	<0.0001	0.13	751.0	−0.11	<0.0001	0.27	151.1	3.61	0.0002	0.30	3135.3	0.35	<0.0001	0.28	953.2

BIC, Bayes Information Criterion; HDL-C, high-density lipoprotein cholesterol; SBP, systolic blood pressure; SD, standard deviation; TG, triglycerides; WC, waist circumference

^1^Results were derived from eight different multivariable linear regression analyses with either manually measured waist circumference or body scanner-based abdominal volume included as independent variable. Parameters of the Metabolic Syndrome were included as single dependent variable. β-coefficients can be interpreted as absolute difference in the parameters of the Metabolic Syndrome, referring to one standard deviation difference in the anthropometric measure. Model adjusted for adjusted for sex, age, and body height (manual measurement).

WC and abdominal volume were highly correlated (r, 0.93; p < 0.0001). When including both in one multivariable adjusted linear regression model, abdominal volume was still significantly associated with TG, HDL-C, and fasting glucose concentrations (p = 0.01, 0.01, and 0.001, respectively), while the associations of WC with these MetS parameters were not statistically significant anymore (p = 0.36, 0.88, and 0.07, respectively). For SBP, the opposite was true (association with WC, p = 0.03, association with abdominal volume, p = 0.65).

### Logistic regression analyses

Overall, there were only small differences between WC and abdominal volume in the discriminative power to detect MetS components (Table 4). For example, a 1-SD higher WC was associated with a 71% higher likelihood (OR and 95% CI: 1.71; 1.28 to 2.31) of the presence of elevated glucose levels, with 24% of the total variance in glucose levels being attributable to WC (R² = 0.24); BIC was 478.3 and c-statistics was 0.787. Contrary, a 1-SD higher abdominal volume was associated with a 91% higher likelihood of elevated glucose levels (1.91; 1.36 to 2.74; R² = 0.24; BIC = 476.9, c-statistics=0.791). There were no significant sex differences in these associations (data not shown).

**Table 4:** Association of manually measured waist circumference and body scanner-based abdominal volume with likelihood of components of the Metabolic Syndrome, total (N = 411)^1^.

	elevated TG^a^				reduced HDL-C^b^				elevated blood pressure^c^				elevated glucose^d^			
anthropometric measure	OR (95% CI)	R²	BIC	c	OR (95% CI)	R²	BIC	c	OR (95% CI)	R²	BIC	c	OR (95% CI)	R²	BIC	c
**manual measure**																
WC, per SD	1.76 (1.29, 2.42)	0.05	384.3	0.677	1.67 (1.08, 2.56)	0.02	222.0	0.658	2.79 (2.03, 3.91)	0.32	440.3	0.839	1.71 (1.28, 2.31)	0.24	478.3	0.787
**body scan measure**																
abdominal volume, per SD	1.76 (1.29, 2.43)	0.05	384.1	0.684	1.65 (1.05, 1.49)	0.02	222.5	0.658	2.67 (1.88, 3.89)	0.30	451.3	0.828	1.91 (1.36, 2.74)	0.24	476.9	0.791

BIC, Bayes Information Criterion; c, c-statistic; HDL-C, high-density lipoprotein cholesterol; OR, odds ratio; SD, standard deviation; TG, triglycerides; WC, waist circumference; 95% CI, 95% confidence interval

^1^Results were derived from eight different multivariable logistic regression analyses with either manually measured waist circumference or body scanner-based abdominal volume included as independent variable. Components of the Metabolic Syndrome were included as single dependent variable. β-coefficients can be interpreted as difference in the likelihood (odds ratio, OR) of metabolic parameters outside the reference range, referring to one standard deviation difference in the anthropometric measure. Model adjusted for sex, age, and body height (manual measurement).

^a^ ≥ 1.7 mmol/L^5^

^b^ men, <1.0 mmol/L; women, <1.3 mmol/L^5^

^c^ systolic blood pressure ≥130 mmHg or diastolic blood pressure ≥85 mmHg (mean of the last two out of three sitting blood pressure measurements) or reported history of hypertension^5^

^d^ ≥ 5.6 mmol/L (or, if information on glucose is missing, HbA1c ≥ 38.8 mmol/mol) or reported history of diabetes^5^.

Finally, we investigated the association of MetS with abdominal volume. A 1-liter higher abdominal volume was associated with a 56% higher likelihood of fulfilling the MetS definition (OR and 95% CI: 1.56; 1.40 to 1.76; R² = 0.35).

### Optimal cutoff analyses

The cutoffs for abdominal volume to differentiate between presence and absence of MetS components were 11.9 L for elevated TG concentrations, 12.8 L for reduced HDL-C concentrations, 12.3 L for elevated blood pressure, 13.1 L for elevated glucose concentrations, and 13.4 L for MetS in men, and were 11.1 L for elevated TG concentrations, 9.7 L for reduced HDL-C concentrations, and were 9.5 L for elevated blood pressure, elevated glucose concentrations, and for MetS, respectively, in women (Supplementary Table S1). The median cutoff was 12.8 L in men and 9.5 L in women, with 63.1% of men and 44.9% of women having an abdominal volume above these limits.

Results for the correlation as well as linear and logistic regression analyses for the association of continuous MetS parameters or dichotomized components, respectively, with BMI and WHtR from manual measurements as well as BS-based AOR and AHtR were not substantially different from the results shown for WC and abdominal volume (Supplementary Tables S2 to S4).

### Additional analyses

When excluding participants with missing MetS parameters instead of imputation, results for the total sample (N = 397) were comparable to the results presented as it was when using WHt²R and AHt²R instead of WHtR and AHtR, respectively (data not shown).

## Discussion

The present study investigated, to what extent abdominal volume determined by a 3D BS can be used as anthropometric risk marker for MetS parameters and components in comparison to the established manually measured WC. We found that the association with TG, HDL-C, and fasting glucose concentrations was slightly stronger and goodness-of-fit with MetS parameters was slightly better for abdominal volume than for WC, while for SBP, we observed the opposite, but differences were small. The discriminative power to assess the frequency of MetS components was similar for abdominal volume and WC. Further, a 1-liter higher abdominal volume was associated with a 56% higher likelihood to have the MetS. Finally, median cutoffs for abdominal volume with the highest differentiating ability of MetS components and MetS were 12.8 L in men and 9.5 L in women. Our data indicate that abdominal volume as assessed using the new BS algorithm is at least as suitable for metabolic characterization as WC. Due to its fast automated determination, it may be a valuable anthropometric parameter to assess metabolic risk in epidemiological studies.

Visceral fat secretes potential intermediary factors related to developing chronic diseases^2–4^. However, a fast direct assessment of visceral fat mass to estimate disease risk, e.g., via magnetic resonance imaging, is not feasible on a large scale. Thus, current practice guidelines recommend measuring WC as an easily measurable surrogate measure of visceral fat to assess abdominal adiposity^22^. However, WC assesses body fat distribution and, thus, morbidity risk insufficiently^3,4^. Directly measured abdominal volume as a 3D measure was supposed to better capture visceral fat accumulation and, thus, metabolic status than WC, which is only a 2-dimensional horizontal measure, or related indices. However, although various BS types are now used in epidemiological studies, to our knowledge, a direct volumetric assessment of abdominal volume using BS does not exist. As there was no built-in algorithm available, we developed and implemented a BS algorithm enabling a fast automated measurement of the 3D abdominal volume.

We found WC and abdominal volume to be significantly correlated with TG, HDL-C, SBP and fasting glucose concentrations, with correlations of abdominal volume to be slightly stronger than for WC, except for SBP. However, overall, differences in correlations between WC and abdominal volume with these parameters were small. This was unexpected, since, as pointed out, abdominal volume was expected to better capture metabolic status than WC. However, the small differences in their correlation with the MetS parameters may partially be explained by the high correlation of both WC and abdominal volume (r = 0.93).

Further, we investigated the strength and goodness-of-fit of the association of MetS parameters with abdominal volume compared to WC. Associations with MetS parameters were slightly stronger and goodness-of-fit was slightly better for abdominal volume than for WC, except for SBP. However, differences between WC and abdominal volume were small, which again may be due to the high correlation between WC and abdominal volume. Given this high correlation, we are aware that results from models including both abdominal volume and WC in one linear regression model have to be interpreted cautiously because of collinearity. However, the aim of this analysis was to assess, which of both measures is associated with MetS parameters, when both are jointly included in the model. We found abdominal volume still to be significantly associated with TG, HDL-C, and glucose concentrations, while WC was not significantly related to these parameters anymore; for SBP the opposite was true. This seems plausible, since visceral adipose tissue, for what abdominal volume is supposedly a better proxy for than WC, intervenes in TG, HDL-C, and glucose metabolism by secreting potential mediators^2^. One may speculate that abdominal volume partly accounts for the association of WC with these MetS parameters, while for SBP, the opposite may be assumed.

Thirdly, we investigated the discriminative power to assess MetS components based on abdominal volume compared to WC. Overall, we found the strength of the association, the proportion of explained variance, as well as the goodness-of-fit criteria to be similar for abdominal volume and WC in detecting MetS components. Given the small differences between WC and abdominal volume observed for the association with continuous MetS parameters, finding no substantial differences for dichotomized parameters seems reasonable. These findings indicate that when categorizing participants using absolute limits in terms of MetS parameters, risk classification based on WC and abdominal volume is likely not substantially different, confirming abdominal volume as suitable anthropometric risk marker.

Our analysis suggests that each 1-liter higher abdominal volume is associated with a 56% higher likelihood of having the MetS. Nevertheless, one should keep in mind that the MetS definition includes elevated WC as one criterion. Therefore, the strong correlation between WC and abdominal volume is likely to have contributed to the high discriminative power (i.e., high OR) of abdominal volume to detect a MetS, which, thus, should be evaluated cautiously.

We also investigated other common manually measured anthropometric parameters (BMI and WHtR) and their BS analogues (AOR and AHtR). For these, results were not substantially different from results shown for WC and abdominal volume. This supports abdominal volume and its ratios as at least as suitable for metabolic characterization as established measures.

Although finding abdominal volume to be only slightly better to assess the metabolic status than WC, 3D BS feature a valuable and efficient anthropometric measurement method for metabolic profiling in epidemiological studies. First, while manual anthropometry is prone to measurement bias^23–26^, BS enable an automated objective, precise, and comprehensive measurement of anthropometry within seconds^10^, which is crucial on a large scale. Since 3D pictures can be stored, data can be re-analysed any time, e.g., with regard to new measures coming up, and individual measures can be developed to identify new metabolic markers. Finally, storage of data and objective evaluation enables a verifiable long-term observation of even small changes in anthropometric markers.

We have BS data of more than 400 participants that encompass a broad spectrum of individual characteristics, e.g., regarding age, anthropometry, and health. We further drew fasting blood samples, enabling sound analyses of metabolic laboratory parameters. All analyses followed standardized protocols and were performed by highly trained personnel. There were no substantial differences between included and excluded participants. Nevertheless, the study population was drawn as convenience sample and was limited to adults, limiting generalizability. Further, we cannot rule out a selection bias, since, generally, participants in epidemiological studies tend to be more health-conscious than the general population^27^. Nevertheless, our study did not aim to be representative of the general population. Thus, further investigation of abdominal volume as anthropometric risk factor is warranted in diverse populations, e.g., including younger or older ages or diseased populations. Our study did not include a ‘gold-standard’ for abdominal volume as determined in our study with the new BS algorithm. Nevertheless, using the same BS in a previous as in the present study, we found good agreement between BS-based overall volume and overall volume based on air-displacement plethysmography as reference method^11^. Since the underlying techniques and technological requirements are the same, we assume a similarly good validity for abdominal as observed for the overall volume. Frequency of elevated glucose was much higher than self-reported numbers of diagnosed diabetes, and was higher than observed for diabetes in the general adult German population^28^. However, it has to be considered that the definition used in the present study to determine glucose status relies on cutoffs corresponding to an impaired fasting glucose status, being lower than those defining diabetes based on international standards, i.e., ≥5.6 mmol/L versus ≥7.0 mmol/L, respectively^5,29^. Finally, it is important to note that our study was cross-sectional, thus, not allowing for investigations of predicting metabolic risk based on abdominal volume.

In conclusion, in our study comparing manually measured WC and BS-based abdominal volume, the association with MetS parameters tended to be slightly stronger and the goodness-of-fit tended to be slightly better for abdominal volume than for WC. However, differences were small. The discriminative power to estimate presence of MetS components was similar for abdominal volume and WC. An optimal cutoff to categorize abdominal volume in terms of metabolic health is 12.8 L in men and 9.5 L in women. Therefore, our data indicate that abdominal volume is at least as suitable for a metabolic characterization as the established manually measured WC.

## Supplementary information

Supplementary Table S1-S4.

## Data Availability

The data that support the findings of this study are available from the authors but restrictions apply to the availability of these data, which were used under license for the current study, and so are not publicly available. Interested researchers (who meet criteria for access to confidential data) may contact the corresponding author of our manuscript for access to the datasets generated and/or analysed during the current study.
