# Supplementary material for: Association of body surface scanner-based abdominal volume with parameters of the Metabolic Syndrome and comparison with manually measured waist circumference
Source: Sci Rep. 2020 Jun 9;10:9324. doi: 10.1038/s41598-020-66095-6 (PMC7283478; doi:10.1038/s41598-020-66095-6)
Supplement: Supplementary file 1 — Supplementary Table S1-S4. [file 41598_2020_66095_MOESM1_ESM.pdf]

Association of body surface scanner-based abdominal volume with parameters of the Metabolic Syndrome and comparison with manually measured waist circumference

Lina Jaeschke[1*], Astrid Steinbrecher[1], Guido Hansen[2], Stefan Sommer[2], Carolin Adler[1], Jürgen Janke[1], Tobias Pischon[1,3,4,5]

[1] Molecular Epidemiology Group, Max Delbrück Center for Molecular Medicine in the Helmholtz Association (MDC), Berlin, Germany

[2] Avalution GmbH, Kaiserslautern, Germany

[3] Charité – Universitätsmedizin Berlin, Berlin, Germany

[4] German Center for Cardiovascular Research (DZHK), partner site Berlin, Berlin, Germany

[5] MDC/BIH Biobank, Max Delbrück Center for Molecular Medicine and Berlin Institute of Health, Berlin, Germany

***Corresponding author**

Lina Jaeschke, Dr. PH, MPH

Molecular Epidemiology Group, Max Delbrück Center for Molecular Medicine in the Helmholtz Association (MDC), Robert-Roessle-Strasse 10, 13125 Berlin, Germany.

Tel. +4930 9406-4597, Fax +4930 9406-4576; e-mail: lina.jaeschke@mdc-berlin.de

Text count: 4130 (excl. title page, abstract, references, figures, and tables); abstract, 199

Supplementary Table S1: Optimal cutoff for body scanner-based abdominal volume with sensitivity, specificity, and Youden Index, total (N=411)[1]

anthropometric measure	elevated TG[a]				reduced HDL-C[b]				elevated blood pressure[c]				elevated glucose[d]				MetS[e]			
	cutoff	sensitivity	specificity	Youden Index[f]	cutoff	sensitivity	specificity	Youden Index[f]	cutoff	sensitivity	specificity	Youden Index[f]	cutoff	sensitivity	specificity	Youden Index[f]	cutoff	sensitivity	specificity	Youden Index[f]
**men** (n=157)																				
**body scan measure**																				
abdominal volume, L	11.9	0.892	0.333	0.225	12.8	0.857	0.380	0.237	12.3	0.837	0.523	0.360	13.1	0.647	0.684	0.331	13.4	0.800	0.713	0.513
**women** (n=254)																				
**body scan measure**																				
abdominal volume, L	11.1	0.576	0.756	0.331	9.7	0.750	0.585	0.335	9.5	0.730	0.734	0.464	9.5	0.675	0.754	0.429	9.5	0.914	0.728	0.643

HDL-C, high-density lipoprotein cholesterol; MetS, metabolic syndrome; TG, triglycerides

[1]Results for were derived from 10 different receiver operating characteristic curves, with the components of the Metabolic Syndrome as outcome and body scanner-based abdominal volume as independent variable. Based on the respective model's intercept and the regression coefficient for abdominal volume, the optimal cutoff was calculated, being evaluated based on the Youden Index.

[a]≥1.7 mmol/L [5]

[b]men, <1.0 mmol/L; women, <1.3 mmol/L [5]

[c]systolic blood pressure ≥130 mmHg or diastolic blood pressure ≥85 mmHg (mean of the last two out of three sitting blood pressure measurements) or reported history of hypertension [5]

[d]≥5.6 mmol/L (or, if information on glucose is missing, HbA1c ≥38.8 mmol/mol) or reported history of diabetes [5]

[e]Metabolic Syndrome was defined as presence of ≥3 of the following five parameters: elevated WC (men, ≥94 cm; women, ≥80 cm), elevated TG concentration (≥1.7 mmol/L), reduced HDL-C concentration (men, <1.0 mmol/L; women, <1.3 mmol/L), elevated blood pressure (mean of the last two out of three sitting blood pressure measurements, systolic blood pressure ≥130 mmHg or diastolic blood pressure ≥85 mmHg, or reported diagnosis of hypertension), elevated glucose concentration (≥5.6 mmol/L or, if information on glucose is missing, HbA1c ≥38.8 mmol/mol, or reported medical history of diabetes) [5].

[f]calculated as sensitivity+specificity-1 [17-19]

Supplementary Table S2: Spearman partial correlation of manually measured and body scanner-based anthropometric measures with parameters of the Metabolic Syndrome, total (N=411)[1]

anthropometric measure	TG, mmol/L		HDL-C, mmol/L		SBP, mmHg		glucose, mmol/L	
	**r**	**p**	**r**	**p**	**r**	**p**	**r**	**p**
**manual measure**								
WC, cm	0.28	<0.0001	-0.28	<0.0001	0.25	<0.0001	0.29	<0.0001
BMI, kg/m²	0.27	<0.0001	-0.29	<0.0001	0.23	<0.0001	0.32	<0.0001
WHtR	0.29	<0.0001	-0.28	<0.0001	0.25	<0.0001	0.29	<0.0001
**body scan measure**								
abdominal volume, L	0.32	<0.0001	-0.32	<0.0001	0.24	<0.0001	0.34	<0.0001
AOR	0.33	<0.0001	-0.29	<0.0001	0.21	<0.0001	0.31	<0.0001
AHtR, L/cm[a]	0.32	<0.0001	-0.32	<0.0001	0.24	<0.0001	0.34	<0.0001

AHtR, abdominal-volume-to-height ratio; AOR, abdominal-to-overall-volume ratio; BMI, body mass index; HDL-C, high-density lipoprotein cholesterol; SBP, systolic blood pressure; TG, triglycerides; WC, waist circumference; WHtR, waist-to-height ratio

[1]Model adjusted for adjusted for age, sex, and body height (manual measurement).

[a]manual height measurement

Supplementary Table S3: Association of manually measured and body scanner-based anthropometric measures with parameters of the Metabolic Syndrome, total (N=411)[1]

	TG, mmol/L				HDL-C, mmol/L				SBP, mmHg				glucose, mmol/L			
	β	p	R^2	BIC	β	p	R^2	BIC	β	p	R^2	BIC	β	p	R^2	BIC
**manual measure**																
WC, per SD	0.19	<0.0001	0.11	759.8	-0.10	<0.0001	0.26	157.2	3.84	<0.0001	0.31	3310.6	0.28	<0.0001	0.25	969.6
BMI, per SD	0.14	<0.0001	0.10	765.8	-0.09	<0.0001	0.26	153.0	2.65	<0.0001	0.30	3318.2	0.25	<0.0001	0.26	966.9
WHtR, per SD	0.18	<0.0001	0.11	760.1	-0.10	<0.0001	0.26	156.7	3.68	<0.0001	0.31	3311.6	0.28	<0.0001	0.25	968.7
**body scan measure**																
abdominal volume, per SD	0.23	<0.0001	0.13	751.0	-0.11	<0.0001	0.27	151.1	3.61	0.0002	0.30	3315.3	0.35	<0.0001	0.28	953.2
AOR, per SD	0.29	<0.0001	0.15	741.3	-0.11	<0.0001	0.25	159.8	4.24	0.0003	0.31	3313.5	0.41	<0.0001	0.29	950.7
AHtR, per SD[a]	0.21	<0.0001	0.13	751.0	-0.11	<0.0001	0.27	150.4	3.41	0.0002	0.30	3314.9	0.34	<0.0001	0.29	950.9

circumference; WHtR, waist-to-height ratio; HDL-C, high-density lipoprotein cholesterol; SBP, systolic blood pressure; SD, standard deviation; TG, triglycerides

[1]Results were derived from 24 different multivariable linear regression analyses with either manually measured or body scanner-based anthropometric measures included as independent variables. Parameters of the Metabolic Syndrome were included as single dependent variable. β-coefficients can be interpreted as absolute difference in the parameters of the Metabolic Syndrome, referring to one standard deviation difference in the anthropometric measure. Model adjusted for sex, age, and body height (manual measurement).

[a]manual height measurement

Supplementary Table S4: Association of manually measured and body scanner-based anthropometric measures with likelihood of components of the Metabolic Syndrome, total (N=411[1]

anthropometric measure	elevated TG[a]				reduced HDL-C[b]				elevated blood pressure[c]				elevated glucose[d]			
	OR (95% CI)	R^2	BIC	c	OR (95% CI)	R^2	BIC	c	OR (95% CI)	R^2	BIC	c	OR (95% CI)	R^2	BIC	c
**manual measure**																
WC, per SD	1.76 (1.29, 2.42)	0.05	384.3	0.677	1.67 (1.08, 2.56)	0.02	222.0	0.658	2.79 (2.03, 3.91)	0.32	440.3	0.839	1.71 (1.28, 2.31)	0.24	478.3	0.787
BMI, per SD	1.57 (1.21, 2.05)	0.05	385.3	0.672	1.54 (1.09, 2.16)	0.02	221.5	0.657	2.07 (1.59, 2.75)	0.30	453.9	0.825	1.59 (1.24, 2.07)	0.24	477.9	0.785
WHtR, per SD	1.75 (1.29, 2.40)	0.05	384.2	0.677	1.68 (1.10, 2.55)	0.02	221.6	0.663	2.75 (2.01, 3.84)	0.32	439.6	0.838	1.68 (1.26, 2.25)	0.24	478.6	0.786
**body scan measure**																
abdominal volume, per SD	1.76 (1.29, 2.43)	0.05	384.1	0.684	1.65 (1.05, 1.49)	0.02	222.5	0.658	2.67 (1.88, 3.89)	0.30	451.4	0.828	1.91 (1.36, 2.74)	0.24	476.9	0.791
AOR, per SD	2.02 (1.40, 2.97)	0.05	382.9	0.685	1.79 (1.02, 3.10)	0.02	223.1	0.652	2.59 (1.81, 3.79)	0.29	456.4	0.822	1.95 (1.37, 2.82)	0.24	477.7	0.789
AHtR, per SD[e]	1.72 (1.29, 2.33)	0.05	383.5	0.686	1.62 (1.08, 2.39)	0.02	222.0	0.662	2.56 (1.84, 3.63)	0.30	449.6	0.829	1.83 (1.34, 2.55)	0.24	476.6	0.790

AHtR, abdominal-volume-to-height ratio; AOR, abdominal-to-overall-volume ratio; Bayes Information Criterion, BIC; BMI, body mass index; c, c-statistics; HDL-C, high-density lipoprotein cholesterol; OR, odds ratio; SD, standard deviation; TG, triglycerides; WC, waist circumference; WHtR, waist-to-height ratio

[1]Results were derived from 24 different multivariable logistic regression analysis with either manually measured or body scanner-based anthropometric measures included as independent variables. Components of the Metabolic Syndrome were included as single dependent variable. β-coefficients can be interpreted as difference in the likelihood (odds ratio) of metabolic parameters outside the reference range, referring to one standard deviation difference of the anthropometric measure. Model adjusted for sex, age, and body height (manual measurement).

[a]≥1.7 mmol/L [5]
[b]men, <1.0 mmol/L; women, <1.3 mmol/L [5]
[c]systolic blood pressure ≥130 mmHg or diastolic blood pressure ≥85 mmHg (mean of the last two out of three sitting blood pressure measurements) or reported history of hypertension[5]
[d]≥5.6 mmol/L (or, if information on glucose is missing, HbA1c ≥38.8 mmol/mol) or reported history of diabetes [5]
[e]manual height measurement
